# Karyotypic Diversity and Evolution in a Sympatric Assemblage of Neotropical Electric Knifefish

**DOI:** 10.3389/fgene.2018.00081

**Published:** 2018-03-19

**Authors:** Adauto L. Cardoso, Julio C. Pieczarka, William G. R. Crampton, Jonathan S. Ready, Wilsea M. B. de Figueiredo Ready, Joseph C. Waddell, Jonas A. de Oliveira, Cleusa Y. Nagamachi

**Affiliations:** ^1^Laboratório de Citogenética, Centro de Estudos Avançados da Biodiversidade, Instituto de Ciências Biológicas, Universidade Federal do Pará, Belém, Brazil; ^2^Conselho Nacional de Desenvolvimento Científico e Tecnológico (CNPq), Brasília, Brazil; ^3^Department of Biology, University of Central Florida, Orlando, FL, United States; ^4^Laboratório de Lepidopterologia e Ictiologia Integrada, Centro de Estudos Avançados da Biodiversidade, Instituto de Ciências Biológicas, Universidade Federal do Pará, Belém, Brazil; ^5^Instituto de Desenvolvimento Sustentável Mamirauá, Tefé, Brazil

**Keywords:** karyotype evolution, chromosome rearrangements, DNA barcode, reproductive isolation, sympatry

## Abstract

Chromosome changes can perform an important role in speciation by acting as post-zygotic reproductive barriers. The Neotropical electric fish genus *Brachyhypopomus* (Gymnotiformes, Hypopomidae) has 28 described species, but cytogenetic data are hitherto available only for four of them. To understand karyotype evolution and investigate the possible role of chromosome changes in the diversification of this genus, we describe here the karyotype of eight species of *Brachyhypopomus* from a sympatric assemblage in the central Amazon basin. We analyzed cytogenetic data in the context of a phylogenetic reconstruction of the genus and known patterns of geographical distribution. We found a strong phylogenetic signal for chromosome number and noted that sympatric species have exclusive karyotypes. Additional insights into the role of chromosome changes in the diversification of *Brachyhypopomus* are discussed.

## Introduction

Reproductive isolation is an essential element to the biological species concept and a driving force in speciation (Dobzhansky, 1937, 1940). Crossings between populations/species that differ in karyotype can generate hybrids with a reduction in fertility associated with mis-segregation of the heterozygous chromosome pairs at meiotic division (hybrid underdominance), and this has the potential to promote sympatric speciation (King, 1993). Moreover, chromosome changes can reinforce the reproductive isolation of incipient species that have entered into secondary contact (White, 1973; King, 1993; Rieseberg, 2001; Navarro and Barton, 2003; Kandul et al., 2007). In other situations, chromosomal differences may accumulate without exerting effects on hybrids, but instead as a result of speciation and diversification in allopatry (Coyne and Orr, 1998). In conjunction with phylogenetic and geographical information, chromosome data [chromosome number, karyotype formula (KF), heterochromatin localization] can therefore be informative of patterns of speciation and diversification (John and Lewis, 1966; Dobigny et al., 2004). Chromosome data can also be useful to elucidate, or support, evolutionary relationships of a group of species, or to solve phylogenetic and taxonomic problems (see for instance Cardoso et al., 2011).

*Brachyhypopomus* Mago-Leccia 1994 is a monophyletic genus of freshwater Neotropical electric fishes (Gymnotiformes) and the most species rich genus in the family Hypopomidae, with 28 described species (Crampton et al., 2016a). The genus is widespread in the Neotropical region (with records from southern Costa Rica and northern Venezuela to Uruguay and northern Argentina) and reaches its highest diversity in Greater Amazonia (the superbasin of the Amazonas and Orinoco basins, and coastal drainages of the Guyanas) (Crampton, 1996; Crampton and Albert, 2006; Crampton et al., 2016a). Greater Amazonia is the center of origin of this genus, with subsequently dispersal events to adjacent basins (Crampton et al., 2016b).

**FIGURE 1:**
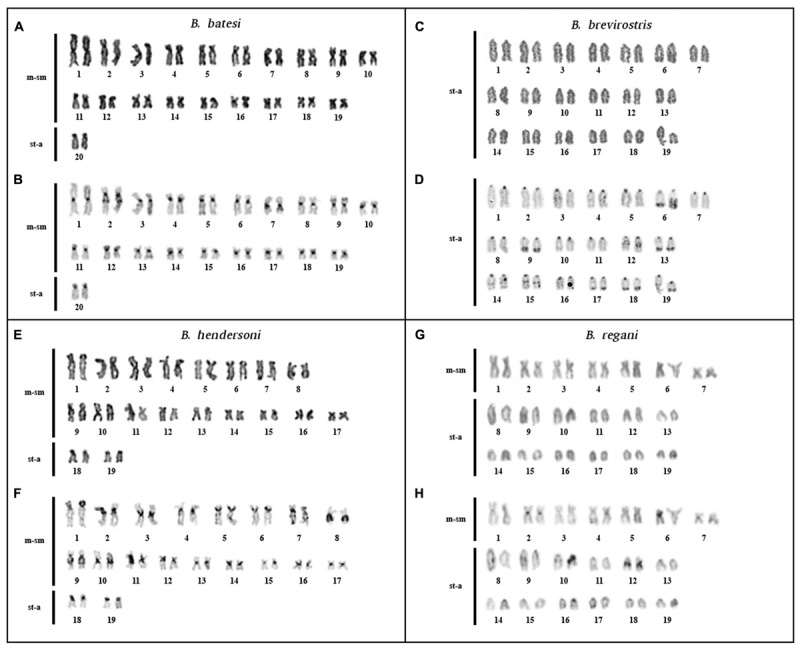
Karyotypes of *Brachyhypopomus batesi* (2*n* = 40), *B. brevirostris* (2*n* = 38), *B. hendersoni* (2*n* = 38), and *B. regani* (2*n* = 38) from Tefé region: elucidated by conventional staining **(A,C,E,G)** and C-banding **(B,D,F,H)**.

In the vicinity of Tefé, in the central Amazon basin (Amazonas State, Brazil), 12 species of *Brachyhypopomus* have been identified from floodplain and *terra firme* streams habitats, representing 43% of diversity in the entire genus (Crampton et al., 2016a, **Figure 1**). This assemblage comprises seven stenotopic floodplain species (*B. belindae*, *B. bennetti*, *B. flavipomus*, *B. hamiltoni*, *B. pinnicaudatus*, and *B. regani* in nutrient-rich whitewaters, and *B. hendersoni* in nutrient-poor blackwaters), two stenotopic *terra firme* stream species (*B. batesi* and *B. sullivani*), and three eurytopic species (*B. beebei*, *B. brevirostris*, and *B. walteri*). This pattern of distributions results in both allotopic and syntopic occurrence of species in the Tefé fauna (Crampton, 2011; Crampton et al., 2016b). Phylogenetic and ecological data indicate sympatry and syntopy of two pair of sister species in this assemblage: *B. beebei* + *B. hamiltoni* (Crampton et al., 2016a,b) and *B. bennetti* + *B. walteri* (Sullivan et al., 2013; Crampton et al., 2016a,b). Other species in the Tefé assemblage have sister species with allopatric distributions. For example, *B. pinnicaudatus* (widespread in the Amazon basin) is sister species to *B. gauderio* (widespread in the Paraná and Patos-Mirim basins of southern South America) (Crampton et al., 2016b). Among the species in the Tefé assemblage, cytogenetic data are previously known only for *B. pinnicaudatus* and *B. flavipomus* (Cardoso et al., 2015a). Additionally, karyotypic information is available for two populations of *B. gauderio* from the Paraná basin (Almeida-Toledo et al., 2000; Mendes et al., 2012).

Here, we provide karyotype descriptions for eight species of *Brachyhypopomus* from the assemblage of Tefé region (**Table 1**) and relate cytogenetic data with phylogenetic relationships and patterns of geographical distribution of these species. We use these data to explore the role of chromosome changes in the diversification of a species-rich Neotropical fish assemblage.

**Table 1:** Samples of the species of *Brachyhypopomus* from Tefé region karyotyped in the present study.

Species		Sample	Habitat type
*Brachyhypopomus batesi*		4 males, 7 females, 1 indeterminate = 12	*Terra firme* stream
*Brachyhypopomus beebei*		9 males, 5 females, 3 indeterminate = 17	Eurytopic
*Brachyhypopomus bennetti*		14 males, 19 females, 1 indeterminate = 34	Whitewater floodplain
*Brachyhypopomus brevirostris*		13 males, 9 females, 7 indeterminate = 29	Eurytopic
*Brachyhypopomus hamiltoni*		3 males, 2 females, 3 indeterminate = 8	Whitewater floodplain
*Brachyhypopomus hendersoni*		9 males, 7 females, 5 indeterminate = 21	Blackwater floodplain
*Brachyhypopomus regani*		2 males, 1 female = 3	Whitewater floodplain
*Brachyhypopomus walteri*		6 males, 6 females = 12	Eurytopic

## Materials and Methods

### Samples

Specimens were captured in the field using a dipnet and electric fish finder (Crampton et al., 2007; Lambert and Crampton, 2010). Identifications followed morphological diagnoses and keys in Crampton et al. (2016b). Specimens were deposited in the ichthyological collections of Museu Paraense Emílio Goeldi and the Instituto de Desenvolvimento Sustentável Mamirauá (Supplementary Table 1).

### Cytogenetic Procedures

Metaphasic chromosomes were obtained following Bertollo et al. (1978). Briefly, 0.025% colchicin was injected in the fish and after 30 min the animal was anesthetized and the kidney was extracted. Kidney cells were treated in hypotonic solution (0.075 M KCl) for 35 min and preserved with Carnoy’s fixative (three ethanol:one acetic acid). Chromosomes were then analyzed by conventional staining (Giemsa 10% for 10 min) and C-banding (HCl 0.2 N for 15 min, Ba(OH)_2_ 5% for 10 s, 2× SSC for 15 min, and Giemsa staining) (Sumner, 1972), and classified following Guerra (1986) in acrocentric, subtelocentric, submetacentric, and metacentric. Levels of karyotype divergence among each possible pair of species were calculated in agreement with Castiglia (2014), including autosomes and sex chromosomes using the following formula: sum of absolute differences in diploid number divided by 2 and the absolute differences in the fundamental number also divided by 2. In order to assess whether karyotype divergence is predicted by phylogenetic distance we performed a clustering procedure using the unweight pair-group method (UPGMA) in the software MEGA 5.0 (Tamura et al., 2011), which is based on Euclidean distance. The matrix of karyotype divergence index is available in Supplementary Table 2.

### COI Barcoding

All samples used in this study were sequenced following protocols established by the Consortium for the Barcode of Life (Ivanova et al., 2005, 2006) and protocols and primers used in Cardoso et al. (2015b). Total genomic DNA was isolated from muscle tissue using DNeasy Tissue Kit (Qiagen), following the manufacturer’s instructions. A portion (661 bp) of the 5’-end of the mitochondrial CO1 gene was amplified by polymerase chain reaction (PCR) using the primers LIICO1F (GATTTTTCTCAACTAACCAYAAAGA) and LIICO1R (ACTTCTGGGTGTCCGAARAAYCARAA). PCR mixes included 6.25 μL of 10% trehalose, 2 μL ultrapure water, 1.25 μL of 10× PCR buffer, 0.625 μL MgCl_2_ (50 mM), 0.125 μL of each primer (0.01 mM), 0.0625 μL of each dNTP (0.05 mM), 0.0625 μL Taq polymerase, and 2.0 μL DNA template. PCR was carried out on a Veriti 96-Well Thermal Cycler (Applied Biosystems, Inc.), under the following conditions: 3 min at 94°C; 40 cycles of 25 s at 94°C, 40 s at 52°C, and 45 s at 72°C; and 5 min at 72°C. Amplified products were checked on 1% agarose gels. PCR products were labeled with BigDye Terminator v3.1 Cycle Sequencing Ready Reaction Kit (ABI) using standard methods and were bidirectionally sequenced on an ABI 3500 DNA Analyzer capillary sequencer following the manufacturer’s instructions. Alignment was made in Geneious R9^1^ (Kearse et al., 2012), mapping new sequences to existing sequences from GenBank and using the consensus of both forward and reverse sequences. All sequences had HQ scores above 88%, no gaps or ambiguous sites were included and no stop codons found. These sequences were submitted to the Barcode of Life Database^2^ under the project “Cytogenetics and Barcoding of Gymnotiformes” (Samples BCG00104–BCG00140).

### Ancestral Chromosome Number Reconstruction

Phylogenetic ancestral character state reconstruction of chromosome number was based on a previously published Bayesian Inference (BI) total evidence species-level tree for the family Hypopomidae, and six outgroup taxa (Crampton et al., 2016a). This tree incorporated 60 morphological characters, approximately 1100 bp of the mitochondrial cytb gene, and approximately 1000 bp of the nuclear rag2 gene; see Crampton et al. (2016b) for methodological details. Using the R package “ape” (Paradis et al., 2004), we pruned species for which the diploid number is unavailable and generated an ultrametric tree with a root length of 1, following Grafen (1989). We then reconstructed ancestral character states for diploid number, which is continuously variable across hypopomids, in a maximum-likelihood framework, using the “phylopars” function in the “Rphylopars” package (Goolsby et al., 2017). Here, we represented ancestral diploid number along branches of the tree with color-maps, using the “contMAP” function in “phytools” package (Revell, 2012). We used the “fastAnc” function in the “phytools” package to find the maximum-likelihood estimate of the ancestral character state for each node. We estimated the strength of phylogenetic signal for diploid number based on Pagel’s lambda statistic (Pagel, 1999) and Blomberg’s *K* statistic (Blomberg et al., 2003), using the “phylosig” function in the R package “phytools” (Revell, 2012).

In addition to performing ancestral character state reconstruction in Rphylopars, we also utilized a second method to infer ancestral character state for chromosome number using the software ChromEvol (Mayrose et al., 2010; Glick and Mayrose, 2014). Here, we utilized the same Crampton et al. (2016b) topology and we adjusted the previous established nomenclature for chromosome changes (Mayrose et al., 2010; Glick and Mayrose, 2014). We used the terms “fusion” and “fission” herein instead of “loss” and “gain,” respectively. In order to find the model that best fits to our data, we performed a first test using “all models” and selected the model with the lowest Akaike information criterion (AIC) value. A second test was then performed with optimized parameters in the best model and no fixed haploid chromosome number. Since ChromEvol reconstructs haploid chromosome number we multiplied the nodal values by 2 to obtain the diploid number.

## Results

### Cytogenetic Data

**FIGURE 2:**
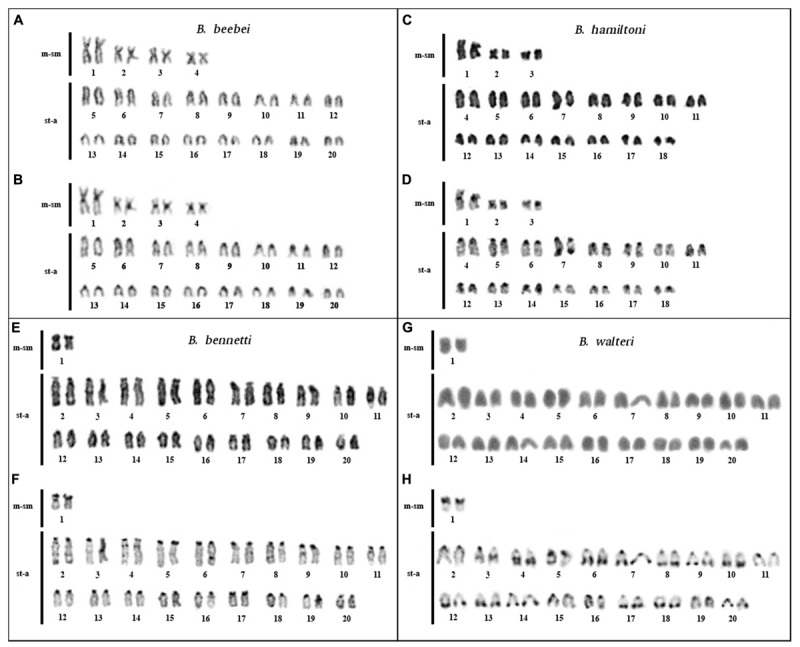
Karyotypes of *Brachyhypopomus beebei* (*n* = 40), *B. hamiltoni* (2*n* = 40), *B. bennetti* (2*n* = 40), and *B. walteri* (2*n* = 40): elucidated by conventional staining **(A,C,E,G)** and C-banding **(B,D,F,H)**.

*Brachyhypopomus batesi*: diploid number (2*n*) = 40 and KF = 38m-sm/2st-a. Constitutive heterochromatin (CH) is localized in the centromeric region, in the interstitial region of 2q, in the proximal region of 7q, and in the distal region of 9p (**Figures 1A,B**). Karyotype differences between males and females were not found.
*Brachyhypopomus brevirostris*: 2*n* = 38 and KF = 38st-a. CH is localized in the centromeric region; in the distal regions of 6p, 9q, 13q, 17q, 18q, and 19q; and in the interstitial regions of 12q, 14q, and 15q (**Figures 1C,D**). Karyotype differences between males and females were not found.
*Brachyhypopomus hendersoni*: 2*n* = 38 and KF = 34m-sm/4st-a. CH localized in the centromeric and in the pericentromeric regions in 1p, 5p, 6p, 14p, and 19p and in the proximal regions of 8q, 9q, 10q, and 11q (**Figures 1E,F**). Karyotype differences between males and females were not found.
*Brachyhypopomus regani*: 2*n* = 38 and KF = 14m-sm/24st-a. CH localized in the centromeric region of all chromosomes and in the interstitial region of 12q (**Figures 1G,H**). Karyotype differences between males and females were not found.
*Brachyhypopomus beebei*: 2*n* = 40, KF = 8m-sm/32st-a. CH localized in the centromeric region of all chromosomes (**Figures 2A,B**). Karyotype differences between males and females were not found.

*Brachyhypopomus hamiltoni*: 2*n* = 36 and KF = 6m-sm/30st-a. CH localized in the centromeric region of all chromosomes and in distal region of 7q (**Figures 2C,D**).
*Brachyhypopomus bennetti*: 2*n* = 40 and KF = 2m-sm/38st-a. CH localized in the centromeric region of all chromosomes and in 1p (**Figures 2E,F**). Karyotype differences between males and females were not found.
*Brachyhypopomus walteri*: 2*n* = 40 and KF = 2m-sm/38st-a. CH localized in the centromeric region, in 1p; in the interstitial region of 3q; and in the distal regions of 4q, 6q, 7q, 8q, 9q, 10q, 12q, 13q, 14q, 17q, 18q, and 20q (**Figures 2G,H**). Karyotype differences between males and females were not found.

**FIGURE 3:**
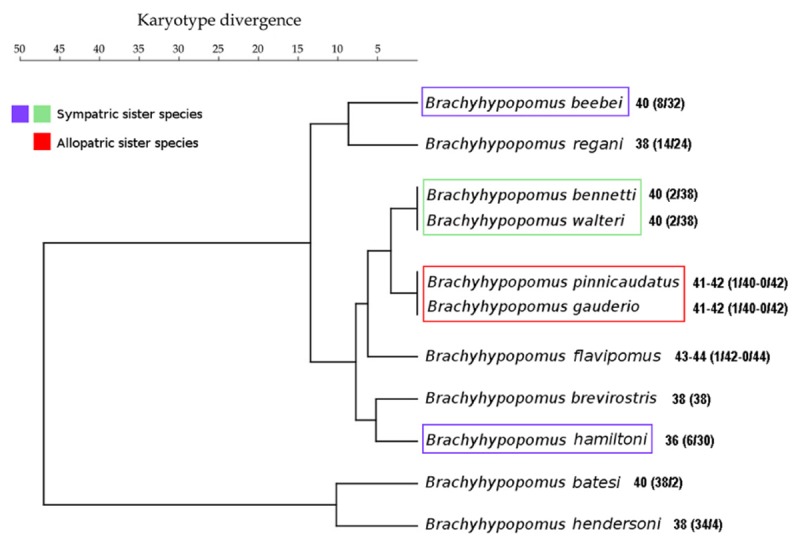
UPGMA phenogram derived from matrix of karyotype divergence index data of species of *Brachyhypopomus* from the assemblage in the Tefé region and *B. gauderio* from Paraná-Paraguay basin. Sisters species pairs identified in the phylogenetic topology of Crampton et al. (2016b) are highlighted.

**FIGURE 4:**
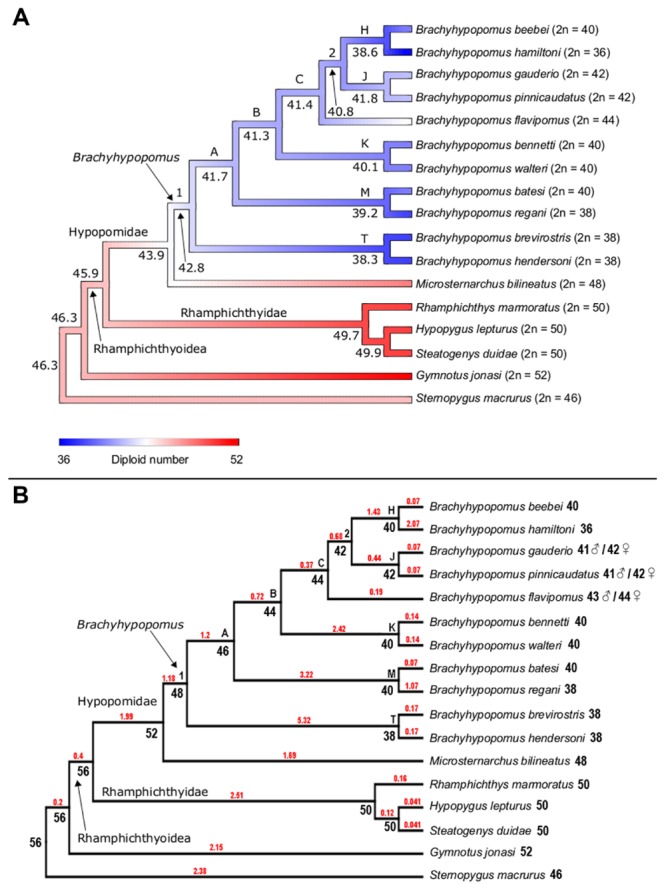
**(A)** Ancestral character state reconstruction for diploid number in the Hypopomidae, Rhamphichthyidae, and outgroups. Color map represents low (blue), intermediate (white), and high (red) values of diploid number, with strongest color intensity representing the extreme low (2*n* = 36) and high (2*n* = 52) values; numbers at the node refer to the reconstructed ancestral diploid number, reported to 1 decimal place; letters on the trees denote well-supported nodes [node supported exceeding 0.88 Bayesian posterior probability (PP)] and numbers report poorly supported nodes (PP < 0.88), following the labeling scheme of Crampton et al. (2016b, see Figure 7). **(B)** Ancestral character state reconstruction for diploid number in the Hypopomidae, Rhamphichthyidae, and outgroups using ChromEvol. Numbers at the node refer to the most probable ancestral haploid number. Red number in branches represents the chromosome loss index.

The clustering of species based on the karyotype divergence index (**Figure 3**) is different to the phylogenetic topology of *Brachyhypopomus* (Crampton et al., 2016b) (**Figure 4**). The phenogram in **Figure 3** also shows that sympatric sister species pair *B. beebei* + *B. hamiltoni* [as identified in the topology of Crampton et al. (2016b)] exhibit more divergent karyotypes than the allopatric sister species *B. gauderio* + *B. pinnicaudatus* (Crampton et al., 2016b). Nonetheless, the sympatric sister species pair *B. bennetti* and *B. walteri* [also as identified in the topology of Crampton et al. (2016b)] do not exhibit divergent karyotypes.

### Ancestral Chromosome Number Reconstruction

Both Rphylopars (**Figure 4A**) and ChromEvol (**Figure 4B**) were used to reconstruct ancestral chromosome number based on the topology of Crampton et al. (2016b) and a dataset of chromosome number. The first method reported highly significant values for Pagel’s lambda statistic (λ = 1.002, *p* = 3.4 × 10^-5^) (Pagel, 1999), and Blomberg’s *K* statistic (*K* = 1.609, *p* = 0.001) (Blomberg et al., 2003), indicating in both cases a strong phylogenetic signal for diploid number (**Figure 4A**). Ancestral chromosome number was inferred in ChromEvol with “Constant Rate with No Duplication,” the best-fitted model for our data (**Table 2**). This model takes into account two parameters for ancestral character reconstruction: “gainConstR” and “lossConstR.” These parameters refer to the occurrence of fission and fusion events, respectively, which are structural chromosome rearrangements that result in changes in chromosome number. “No Duplication” model indicates the absence of polyploidization events during evolution, and this does not exclude the occurrence of gain of chromosome segments. Chromosome fusions were the only events observed (total loss = 36.14) (**Figure 4B**).

**Table 2:** AIC values of the run with all models of karyotype evolution.

Model	Log-likelihood	AIC
CONST_RATE	-29.83	65.66
CONST_RATE_DEMI	-29.84	65.67
CONST_RATE_DEMI_EST	-29.83	67.66
**CONST_RATE_NO_DUPL**	-**29.83**	**63.66**
LINEAR_RATE	-29.82	69.64
LINEAR_RATE_DEMI	-29.82	69.64
LINEAR_RATE_DEMI_EST	-30.1	72.2
LINEAR_RATE_NO_DUPL	-29.82	67.64

*Constant rate with no duplication (in bold) had the minor AIC values, which indicate the best-fitting model for our data*.

Rphylopars and ChromEvol provided congruent reconstructions of ancestral chromosome numbers in the nodes that correspond to the *Brachyhypopomus* groups B, C, 2, H, J, K, M, and T, as well as in the nodes that correspond to Rhamphichthyidae and Steatogeni (**Figure 4** and **Table 3**). However, there were incongruences at the following nodes: Rhamphichthyoidea (the superfamily comprising Rhamphichthyidae and Hypopomidae), Hypopomidae, and *Brachyhypopomus*.

**Table 3:** Comparison of the two methods used to reconstruct ancestral chromosome number.

Node	RPhylo tools				ChromEvol			
	Ancestral character state estimate	Variance	95% CI lower	95% CI upper	Ancestral character state estimate
1	46.260	6.431	41.289	51.230	56/58
2	46.276	5.409	41.717	50.834	56/58
3	45.910	4.892	41.575	50.246	56/54
4	43.860	3.835	40.022	47.699	52/50
5	42.800	3.520	39.123	46.478	48/50
6	41.691	3.133	38.222	45.161	46/48
7	41.300	2.619	38.128	44.472	44/46
8	41.381	1.917	38.668	44.095	44/46
9	40.767	1.559	38.320	43.215	42/44
10	38.553	0.759	36.846	40.261	40/42
11	41.753	0.759	40.046	43.461	42/44
12	40.118	0.813	38.351	41.886	40/42
13	39.179	0.827	37.397	40.961	40/42
14	38.253	0.835	36.462	40.043	38/40
15	49.727	1.415	47.396	52.059	50/52
16	49.909	0.738	48.226	51.592	50/52

*The first and the second most probable chromosome number determined by ChromEvol were multiplied by 2 (diploid number)*.

## Discussion

### Karyotypic Diversity in *Brachyhypopomus*

The cytogenetic data generated in this study, in combination with previous studies (Almeida-Toledo et al., 2000; Mendes et al., 2012; Cardoso et al., 2015a), reveal interspecific karyotypic divergence for the genus *Brachyhypopomus* in general, and for the Tefé assemblage in particular (**Table 4**). These differences are reflected in the diploid number (2*n*), which can result from chromosome fusion/fission events (Milhomem et al., 2008). Despite 2*n* divergence, some species share 2*n* values (e.g., *B. brevirostris* and *B. hendersoni*), but they exhibit different KFs, which result from events that modify the chromosome morphology, but that do not change the 2*n*, such as pericentric inversions, translocations of chromosome segments, and centromere repositioning (White, 1973; Montefalcone et al., 1999). Therefore, various types of chromosome rearrangements appear to be involved in the karyotype diversification in *Brachyhypopomus*, as has previously documented at both interspecific and intraspecific levels in other Gymnotiformes, e.g., *Gymnotus* and *Eigenmannia* (Fernandes-Matioli and Almeida-Toledo, 2001; Milhomem et al., 2008, 2012; Silva et al., 2009; Nagamachi et al., 2010, 2013).

**Table 4:** Compilation of the cytogenetic data of the species of *Brachyhypopomus*.

Species	Basin/area	2*n*	KF	SCS	CH
*Brachyhypopomus hamiltoni*	Tefé region	36	6m-sm/30st-a	Unidentified	Centromeric; distal in 7q


*Brachyhypopomus brevirostris*	Tefé region	38	38st-a	Unidentified	Centromeric; distal in 6p, 9q, 13q, 17q, 18q, and 19q; interstitial in 12q, 14q, and 15q


*Brachyhypopomus hendersoni*	Tefé region	38	34m-sm/4st-a	Unidentified	Centromeric; pericentromeric; 1p, 5p, 6p, 14p, 19p; proximal in 8q, 9q, 10q, 11q


*Brachyhypopomus regani*	Tefé region	38	14m-sm/24st-a	Unidentified	Centromeric; interstitial in 12q


*Brachyhypopomus batesi*	Tefé region	40	38m-sm/2st-a	Unidentified	Centromeric; interstitial in 2q; proximal in 7q; distal in 9p


*Brachyhypopomus beebei*	Tefé region	40	8m-sm/32st-a	Unidentified	Centromeric


*Brachyhypopomus bennetti*	Tefé region	40	2m-sm/38st-a	Unidentified	Centromeric; 1p


*Brachyhypopomus walteri*	Tefé region	40	2m-sm/38st-a	Unidentified	Centromeric; 1p; interstitial in 3q; distal in 4q, 6q, 7q, 8q, 9q, 10q, 12q, 13q, 14q, 17q, 18q, 20q


*Brachyhypopomus pinnicaudatus*^∗^	Tefé region	41bbb/42ccc	1m-sm/40st-abbb 42st-accc	X_1_X_1_X_2_X_2_/X_1_X_2_Y	Centromeric; short bands in 1q


*Brachyhypopomus gauderio*^∗∗^	Paraná-Paraguay basin	41bbb/42ccc	1m-sm/40st-abbb 42st-accc	X_1_X_1_X_2_X_2_/X_1_X_2_Y	Centromeric


*Brachyhypopomus flavipomus*^∗^	Tefé region	43bbb/44ccc	1m-sm/42st-abbb 44st-accc	X_1_X_1_X_2_X_2_/X_1_X_2_Y	Centromeric; distal in 15q

^∗^*Karyotypic information of these species was obtained from Cardoso et al. (2015a); ^∗∗^Chromosomal data were obtained from Almeida-Toledo et al. (2000) and Mendes et al. (2012). 2*n*, diploid number; KF, karyotypic formula; SCS, sexual chromosome system; CH, constitutive heterochromatin*.

Although several species of *Brachyhypopomus* share the same 2*n* but different KFs, two pairs of sister species share both 2*n* and KF: *B. bennetti* + *B. walteri* (sympatric in Tefé region) and *B. pinnicaudatus* + *B. gauderio* (allopatric). The latter pair also share a putatively homologous multiple sex chromosome system (Cardoso et al., 2015a). Despite the apparent karyotypic conservation in these two sister species pairs, there is divergence in the localization of the CH between sister species. This divergence is greater between *B. bennetti* and *B. walteri* than between *B. pinnicaudatus* and *B. gauderio* (**Figure 2**), suggesting that *B. bennetti* and *B. walteri* may be reproductively isolated by chromosome changes. We report divergent patterns of CH in all species of *Brachyhypopomus*, ranging from species with exclusive centromeric CH to species with extra heterochromatic blocks in proximal, interstitial, and distal regions of the chromosomes (**Figures 1**, **2** and **Table 4**). CH variation can be a result of the fast evolution of repetitive sequences, which are important component of CH and are involved in events of chromosome rearrangements (Dimitri et al., 2009). The association of heterochromatin variation with 2*n* and KF suggests the possible involvement of the CH in the occurrence of chromosomal rearrangements, as observed in *Drosophila* (Yoon and Richardson, 1978).

The interspecific karyotypic divergence found in *Brachyhypopomus* reveals that sympatric species exhibit unique karyotypes that are diagnostic of species identity.

### Karyotypic Evolution in *Brachyhypopomus*

**FIGURE 5:**
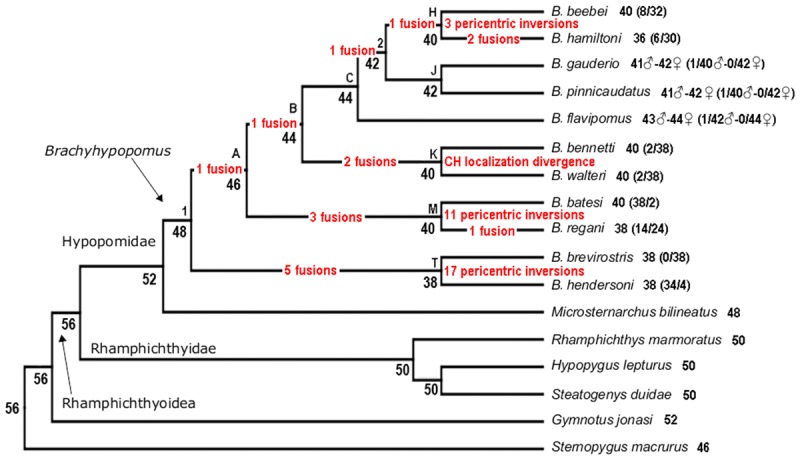
Phylogenetic tree (Crampton et al., 2016b) showing chromosome changes in *Brachyhypopomus* species based on character state reconstruction for chromosome numbers, karyotype formulas, and constitutive heterochromatin localization.

Two methods for reconstructing ancestral chromosome number (Rphylopars and ChromEvol) reveal a strong phylogenetic pattern of chromosome number reduction attending diversification in *Brachyhypopomus*. Chromosome fusions are evidently the events that promote this reduction (**Figure 4**). Furthermore, based on chromosome morphologies and the ancestral chromosome numbers provided by ChromEvol, we propose the set of chromosome rearrangements involved in karyotype evolution (**Figure 5**). From the ancestral condition of *Brachyhypopomus* (2*n* = 48), clade A is subject to one chromosome fusion and clade T is subject to five fusions. From node T, *B. brevirostris* and *B. hendersoni* diverge by 17 pericentric inversions. From node A, node B has one fusion and node M has three fusions. From node M, *B. regani* accumulates one fusion and is divergent from *B. batesi* by 11 pericentric inversions. From node B, node C maintains the chromosome number and node K has two fusions. From node K, *B. bennetti* and *B. walteri* retain a conserved diploid number and are divergent by CH localization. From node C, *B. flavipomus* retains the chromosome number and node 2 accumulates one fusion. From node 2, node J keeps the chromosome number and node H exhibits one fusion. From node J, *B. gauderio* and *B. pinnicaudatus* diverge without chromosome change. From node H, *B. hamiltoni* accumulates two fusions and is divergent from *B. beebei* by three pericentric inversions. However, we were unable to identify rearrangements that do not change chromosome number and morphology, such as paracentric inversions and reciprocal translocations. The numbered nodes in **Figures 4A,B** denote clades with poor nodal support (posterior probabilities <0.88, versus >0.88 in all other nodes) (Crampton et al., 2016b), and we acknowledge that this may affect the accuracy of ancestral chromosome reconstructions, as well as the chromosome rearrangements proposed above.

Chromosome fusions were identified herein as important events in the diversification of *Brachyhypopomus*, and have also been observed in *Microsternarchus bilineatus* (de Jesus et al., 2016; Batista et al., 2017). In contrast, fusions have not been registered among species of Rhamphichthyidae (the sister group to Hypopomidae), which exhibits a karyotype evolution characterized by 2*n* conservation (50 chromosomes) and chromosome inversions (Cardoso et al., 2011; Mendes et al., 2012; Silva et al., 2013). These finds suggest that chromosomal fusions were an important force in the diversification of Hypopomidae. Nonetheless, more hypopomid species need to be cytogenetically analyzed to confirm this hypothesis.

### Karyotypic Diversity, Reproductive Isolation, and Speciation in *Brachyhypopomus*

The interspecific karyotypic divergences found in the Tefé *Brachyhypopomus* assemblage suggest that post-zygotic reproductive isolation between these species may have been important during their evolution, although understanding the role of chromosome changes in promoting reproductive isolation during the process of speciation is challenging. Correlation of cytogenetic data with the phylogeny and the geographical distribution of these species may nonetheless be informative of how post-zygotic barriers can arise. The phylogenetic reconstruction of *Brachyhypopomus* species by Crampton et al. (2016a) indicates that the Tefé assemblage is non-monophyletic and comprises species with wide geographical distributions, some of which are closely related to species from other regions (e.g., *B. pinnicaudatus*). This indicates that this assemblage does not represent a localized radiation (i.e., “species flock”), but instead arose from dispersal assembly of species that originated by allopatric speciation elsewhere, a pattern that is as well described for Neotropical fish fauna (Albert et al., 2011; Crampton, 2011). This further implies that the accumulation of chromosomal differences between most species in this assemblage did not occur *in situ*, but instead is an incidental product of divergences in allopatry. The Amazon basin has passed through a range of geological process (orogeny, uplifting, erosion, and river capture), which generated opportunities for allopatric speciation followed by secondary contacts (Crampton, 2011). In some cases, populations or incipient species that diverged in allopatry and then come into secondary contact may have become reproductively isolated as a result of karyotypic differences that negatively affect the fitness or the sterility of hybrids (White, 1973; King, 1993; Noor et al., 2001; Rieseberg, 2001; Navarro and Barton, 2003). By these means even karyotype differences acquired in allopatry may have contributed to the maintenance of nascent species. Nonetheless, the sympatric occurrence of sister species (*B. bennetti* + *B*. *walteri* and *B. beebei* + *B. hamiltoni*) provides some evidence for geographically localized speciation.

The data reported in **Figure 3** show that the allopatric sister species *B. pinnicaudatus* + *B. gauderio* exhibit a karyotype divergence of 0% while the sympatric sister species *B. beebei* + *B. hamiltoni* exhibit a divergence of 2%. This pattern, which indicates that sympatric sister species are more divergent than allopatric sister species, is consistent with the role of chromosome changes in promoting reproductive isolation and has also been found in the butterfly genus *Agrodiaetus* (Kandul et al., 2007), as well as in rodents (Castiglia, 2014). Unlike in the sympatric sister pair *B. beebei* + *B. hamiltoni*, the pattern in the sympatric species pair *B. bennetti* + *B. walteri*, which exhibits a karyotype divergence of 0%, is (at least superficially) inconsistent with the involvement of chromosome rearrangements in reproductive isolation. However, a limitation of the method of measuring karyotype divergence is that it only considers chromosome rearrangements that change 2*n* and KF, while excluding other types of rearrangements that cannot be detected by conventional chromosome staining and C-banding. Indeed, more refined methods (e.g., using fluorescence *in situ* hybridization) reveal that the number of rearrangements can be greater than supposed by classic cytogenetic methods, as previously observed between two cryptic karyomorphs of *Gymnotus carapo* (Nagamachi et al., 2010). Moreover, the method of measuring karyotype divergence we employ herein does not take into account variation in the CH location, which is very divergent between *B. bennetti* and *B. walteri* (**Figures 2F,H**), and which may play a role in reproductive isolation (Ferree and Barbash, 2009; Hughes and Hawley, 2009). For example, a heterochromatin block on the paternally inherited X chromosome has a lethal effect in female hybrids of *Drosophila simulans* females and *D. melanogaster* males due to abnormal chromosome segregation during anaphase of mitotic divisions 10–13 in embryos, when heterochromatin is first established (Ferree and Barbash, 2009). A rearranged X chromosome without this CH block segregates normally in female hybrids, but a translocation of this block to the Y chromosome promotes the same deleterious effect in male hybrids. Alternatively, it is possible that variations in CH distribution between *Brachyhypopomus* species does not play a role in reproductive isolation, but instead indicates greater activity of repetitive sequences, such as transposable elements, which can result in chromosome rearrangements (Dimitri et al., 2009). To summarize, it is possible that karyotypic changes have played an important role in promoting post-zygotic reproductive isolation in some *Brachyhypopomus* species of the Tefé region, either by chromosome rearrangement or heterochromatin effect, and likewise either during secondary contact or during speciation in sympatry.

According to Albert and Crampton (2005) a combination of processes including speciation, extinction, immigration, and ecological factors allowing coexistence in sympatry, contribute to the formation of local assemblages of Gymnotiformes. This study, as well as others that have identified karyotypic divergence among sympatric species of Gymnotiformes from the Tefé region (Milhomem et al., 2012) or elsewhere (Lacerda and Maistro, 2007; Margarido et al., 2007; Milhomem et al., 2008), leading us to the conclusion that karyotypic differences may play an important role in the origins and maintenance of community diversity in Neotropical fish fauna.

## Ethics Statement

This study was carried out in accordance with the recommendations of Comitê de Ética Animal da Universidade Federal do Pará with written informed consent from all subjects. All subjects gave written informed consent in accordance with the Declaration of Helsinki. The protocol was approved by the Comitê de Ética Animal da Universidade Federal do Pará (Permit 68/2015).

## Author Contributions

AC, JP, WC, and CN provided the substantial contributions to the conception of the work. AC, JP, and CN performed the acquisition, analysis, and interpretation of cytogenetic data. AC, WC, JW, and JdO performed the acquisition, analysis, and interpretation of morphological data. AC, WC, JR, WdFR, and JW performed the acquisition, analysis, and interpretation of molecular data. AC, JP, WC, JR, WdFR, JW, JdO, and CN involved in writing the draft of the work or revised it critically. AC, JP, WC, JR, WdFR, JW, JdO, and CN provided the final approval of the version to be published.

## Conflict of Interest Statement

The authors declare that the research was conducted in the absence of any commercial or financial relationships that could be construed as a potential conflict of interest.

## Footnotes

1 http://www.geneious.com

2 http://www.boldsystems.org/index.php/databases
