# Supplementary material for: Karyotypic Diversity and Evolution in a Sympatric Assemblage of Neotropical Electric Knifefish
Source: Front Genet. 2018 Mar 19;9:81. doi: 10.3389/fgene.2018.00081 (PMC5867350; doi:10.3389/fgene.2018.00081)
Supplement: Supplementary file 1 [file Table_1.docx]

**Supplementary Table 1.** Coordinates of *Brachyhypopomus* species samples studied in the present work.

| **Species** | **Voucher number/Locality** |
| --- | --- |
| *Brachyhypopomus batesi* | MPEG 27124 (03°25’56”S, 064º40’43.5’’W) |
| *Brachyhypopomus beebei* | MPEG 22741 (03°07’19.9”S, 064°48’06.0”W); MPEG 22742 (03°03’46.6”S, 064°49’58.8”W ); IDSMIctio 739 (03°00'56.2"S, 064°51'12.3"W); IDSMIctio 740 (03°07'32.5"S, 064°46'47.3"W); IDSMIctio 800 (03°00'56.2"S, 064°51'12.3"W); IDSMIctio 802 (03°00'56.2"S, 064°51'12.3"W); IDSMIctio 818; IDSMIctio 822 (03°00'56.2"S, 064°51'12.3"W); IDSMIctio 823 (03°00'56.2"S, 064°51'12.3"W); IDSMIctio 829 (03°00'56.2"S, 064°51'12.3"W) |
| *Brachyhypopomus bennetti* | MPEG 22745 (02°59’38”S, 064°54’25.74”W); MPEG 22746 (03°03’46.6”S, 064°49’58.8”W); IDSMIctio 730 (03°01'41.8"S, 064°51'16.6"W); IDSMIctio 749 (03°06’12.4’’S, 064°47'55.4"W); IDSMIctio 826 (03°01'41.8"S, 064°51'16.6"W); IDSMIctio 827 (03°01'41.8"S, 064°51'16.6"W); IDSMIctio 837 (03°01'41.8"S, 064°51'16.6"W); IDSMIctio 840 (03°01'41.8"S, 064°51'16.6"W); IDSMIctio 866 (03°01'41.8"S, 064°51'16.6"W) |
| *Brachyhypopomus brevirostris* | MPEG 22738 (02°59’38”S, 064°54’25.74”W); MPEG 22739 (03°24’19.2”S, 064°43’53.6”W); IDSMIctio 743 (03°00'56.2"S, 064°51'12.3"W); IDSMIctio 774 (03°06’12.4”S, 064°47'55.4"W); IDSMIctio 791 (03º10'50.7''S, 064º45'47,1''W); IDSMIctio 807 (03º10'50.7''S, 064º45'47,1''W); IDSMIctio 811 (03º10'50.7''S, 064º45'47,1''W); IDSMIctio 813 (03º10'50.7''S, 064º45'47,1''W); IDSMIctio 816 (03º10'50.7''S, 064º45'47,1''W); IDSMIctio 819 (03º10'50.7''S, 064º45'47,1''W); IDSMIctio 841 (03º10'50.7''S, 064º45'47,1''W); IDSMIctio 847 (03º10'50.7''S, 064º45'47,1''W); IDSMIctio 848 (03º10'50.7''S, 064º45'47,1''W); IDSMIctio 870 (03º10'50.7''S, 064º45'47,1''W) |
| *Brachyhypopomus hamiltoni* | MPEG 22744 (03°03’46.6”S, 064°49’58.8W); MPEG 27114 (03º16'03.1"S, 064º40'50.3"W); MPEG 27115 (03°02’57.5”S, 064°50’56.9”W); MPEG 27125 (03°25’56”S; 064º40’43.5W’’) |
| *Brachyhypopomus hendersoni* | IDSMIctio 828 (03º 20' 29.4''S, 064º 40' 40.7''W); IDSMIctio 846 (03º 20' 29.4''S, 064º 40' 40.7''W); IDSMIctio 850 (03º 20' 29.4''S, 064º 40' 40.7''W); IDSMIctio 853 (03º 20' 29.4''S, 064º 40' 40.7''W); IDSMIctio 855 (03º 20' 29.4''S, 064º 40' 40.7''W); IDSMIctio 865 (03º 20' 29.4''S, 064º 40' 40.7''W) |
| *Brachyhypopomus regani* | MPEG 27112 (02º48.43'S; 65º04.55'W) |
| *Brachyhypopomus walteri* | MPEG 22747 (02°59’38”S, 64°54’25.74”W); MPEG 22748 (03°03’46.6”S, 064°49’58.8”W); IDSMIctio 729 (03°01’41.8”S, 064°51'16.6"W); IDSMIctio736 (03°00'56.2"S, 064°51'12.3"W); IDSMIctio741 (03°07'32.5"S, 064°46'47.3"W); IDSMIctio748 (03°07'32.5"S, 064°46'47.3"W); IDSMIctio830 (03°07'32.5"S, 064°46'47.3"W); IDSMIctio842(03°07'32.5"S, 064°46'47.3"W); IDSMIctio854 (03°07'32.5"S, 064°46'47.3"W); IDSMIctio 859 (03°07'32.5"S, 064°46'47.3"W) |
