# Supplementary material for: Karyotypic Diversity and Evolution in a Sympatric Assemblage of Neotropical Electric Knifefish
Source: Front Genet. 2018 Mar 19;9:81. doi: 10.3389/fgene.2018.00081 (PMC5867350; doi:10.3389/fgene.2018.00081)
Supplement: Supplementary file 2 [file Table_2.docx]

**Supplementary Table 2.** Karyotype divergence index matrix of the species of *Brachyhypopomus*. 2n: diploid number; FN: fundamental number; A: *B. beebei*; B: *B. bennetti*; C: *B. brevirostris*; D: *B. walteri*; E: *B. pinnicaudatus*; F: *B. batesi*; G: *B. flavipomus*; H: *B. hamiltoni*; I: *B. hendersoni*; J: *B. regani*; K: *B. gauderio*.

|  | **2n** | **FN** | **A** | **B** | **C** | **D** | **E** | **F** | **G** | **H** | **I** | **J** | **K** |
| --- | --- | --- | --- | --- | --- | --- | --- | --- | --- | --- | --- | --- | --- |
| **A** | 40 | 48 | 0 |  |  |  |  |  |  |  |  |  |  |
| **B** | 40 | 42 | 3 | 0 |  |  |  |  |  |  |  |  |  |
| **C** | 38 | 38 | 6 | 3 | 0 |  |  |  |  |  |  |  |  |
| **D** | 40 | 42 | 3 | 0 | 3 | 0 |  |  |  |  |  |  |  |
| **E** | 42 | 42 | 4 | 1 | 4 | 1 | 0 |  |  |  |  |  |  |
| **F** | 40 | 78 | 15 | 18 | 21 | 18 | 19 | 0 |  |  |  |  |  |
| **G** | 44 | 44 | 4 | 3 | 6 | 3 | 2 | 19 | 0 |  |  |  |  |
| **H** | 36 | 42 | 5 | 2 | 3 | 2 | 3 | 20 | 5 | 0 |  |  |  |
| **I** | 38 | 72 | 13 | 16 | 17 | 16 | 17 | 4 | 17 | 16 | 0 |  |  |
| **J** | 38 | 52 | 3 | 6 | 7 | 6 | 7 | 14 | 7 | 6 | 10 | 0 |  |
| **K** | 42 | 42 | 4 | 1 | 4 | 1 | 0 | 19 | 2 | 3 | 17 | 7 | 0 |
